# A Resting ECG Screening Protocol Improved with Artificial Intelligence for the Early Detection of Cardiovascular Risk in Athletes

**DOI:** 10.3390/diagnostics15040477

**Published:** 2025-02-16

**Authors:** Luiza Camelia Nechita, Dana Tutunaru, Aurel Nechita, Andreea Elena Voipan, Daniel Voipan, Anca Mirela Ionescu, Teodora Simina Drăgoiu, Carmina Liana Musat

**Affiliations:** 1 Faculty of Medicine and Pharmacy, ‘Dunarea de Jos’ University of Galati, 800008 Galati, Romania; luiza.nechita@ugal.ro (L.C.N.); dana.tutunaru@ugal.ro (D.T.); aurel.nechita@ugal.ro (A.N.); carmina.musat@ugal.ro (C.L.M.); 2 Faculty of Automation, Computers, Electrical Engineering and Electronics, ‘Dunarea de Jos’ University of Galati, 800008 Galati, Romania; 3 Faculty of Medicine, University of Medicine and Pharmacy ‘Carol Davila’, 020022 Bucharest, Romania; anca.ionescu@umfcd.ro (A.M.I.); teodora-simina.ionescu@drd.umfcd.ro (T.S.D.); 4 The National Institute of Sports Medicine, 022103 Bucharest, Romania; 5 European Federation of Sports Medicine Association, CH-1007 Lausanne, Switzerland

**Keywords:** artificial intelligence, cardiovascular screening, electrocardiogram (ECG), athlete safety, risk classification, machine learning, statistics, predictive analytics, sports medicine, risk prevention

## Abstract

**Background/Objectives**: This study aimed to evaluate an artificial intelligence (AI)-enhanced electrocardiogram (ECG) screening protocol for improved accuracy, efficiency, and risk stratification across six sports: handball, football, athletics, weightlifting, judo, and karate. **Methods:** For each of the six sports, resting 12-lead ECGs from healthy children and junior athletes were analyzed using AI algorithms trained on annotated datasets. Parameters included the QTc intervals, PR intervals, and QRS duration. Statistical methods were used to examine each sport’s specific cardiovascular adaptations and classify cardiovascular risk predictions as low, moderate, or high risk. **Results:** The accuracy, sensitivity, specificity, and precision of the AI system were 97.87%, 75%, 98.3%, and 98%, respectively. Among the athletes, 94.54% were classified as low risk and 5.46% as moderate risk with AI because of borderline abnormalities like QTc prolongation or mild T-wave inversions. Sport-specific trends included increased QRS duration in weightlifters and low QTc intervals in endurance athletes. **Conclusions:** The statistical analyses and the AI-ECG screening protocol showed high precision and scalability for the proposed athlete cardiovascular health risk status stratification. Additional early detection research should be conducted further for diverse cohorts of individuals engaged in sports and explore other diagnostic methods that can help increase the effectiveness of screening.

## 1. Introduction

The cardiovascular health of young athletes is a strong predictor of both the well-being and the performance of the athlete [1]. While such physical activity is beneficial in evoking physiological adaptations, it may also unmask subclinical conditions that could expose athletes to adverse events [2]. Sudden cardiac death (SCD), however rare, is one of the biggest fears in competitive sports [3]. Thus, the early identification of cardiovascular risks through routine screening is crucial for ensuring athlete safety and longevity in sports [4]. Recent studies indicate that the early detection of cardiovascular abnormalities in athletes can significantly reduce the incidence of sudden cardiac events [5,6,7,8,9]. A comprehensive screening protocol incorporating advanced predictive analytics could enhance diagnostic precision and help differentiate physiological adaptations from pathological conditions [10].

The electrocardiogram (ECG) is the most popular tool for cardiovascular evaluation and can detect electrical and structural abnormalities that may not be visible otherwise. While ECG is a widely used diagnostic tool, its interpretation in athletes presents unique challenges due to the physiological adaptations induced by regular training, which can mimic pathological conditions, leading to frequent misclassification [11]. There is a particular difficulty in athletes since the ECG changes that occur with exercise are similar to those in disease (e.g., sinus bradycardia, early repolarization) [12]. For instance, endurance athletes commonly exhibit increased QRS voltage due to left ventricular hypertrophy, which could be mistakenly identified as hypertrophic cardiomyopathy if proper screening criteria are not applied [13]. Similarly, repolarization abnormalities observed in strength athletes may be misinterpreted as ischemic changes, necessitating further diagnostic evaluation [14,15].

Traditional manual interpretation, based on criteria like the Seattle guidelines, is repetitive and subjective, especially when evaluating borderline results [16,17]. Furthermore, inter-observer variability in ECG interpretation remains a significant issue, as studies have shown that even experienced cardiologists may have differing opinions on borderline cases [6,7]. This variability underscores the need for more standardized and objective approaches, such as AI-assisted ECG analysis, which can reduce inconsistencies and improve diagnostic reliability [8,9].

Artificial intelligence (AI) is revolutionary in this regard [18]. Recent advancements in AI-driven ECG interpretation have demonstrated significant improvements in diagnostic accuracy, especially in distinguishing between physiological adaptations and true pathological findings. AI-based models utilize deep learning algorithms trained on large datasets, enabling the more precise and scalable classification of cardiovascular risks [19].

This study proposes a novel AI-based ECG screening approach for detecting cardiovascular abnormalities in a population of young Romanian athletes, engaged in handball, football, athletics, weightlifting, judo, and karate. The system analyzes ECG data, classifies the outcomes, and recommends the level of risk (low, moderate, or high) for each athlete based on the analysis of key parameters.

For classification, we implemented a Random Forest (RF) classifier, which demonstrated strong predictive performance in distinguishing between physiological adaptations and potential cardiovascular risks. The model was trained using a dataset of annotated ECG recordings, incorporating key features such as the QRS duration, QTc interval, PR interval, and heart rate. Hyperparameter tuning was conducted using grid search optimization to enhance performance. The RF model was selected for its robustness in handling complex interactions between features and its ability to provide interpretable feature importance rankings. To address class imbalance, a combination of the Synthetic Minority Over-sampling Technique (SMOTE) and class weighting was applied during training, ensuring improved sensitivity for detecting moderate-risk cases. The model achieved an accuracy of 97.87%, specificity of 98.3%, and sensitivity of 75%, making it a highly effective tool for athlete cardiovascular screening.

Furthermore, AI enhances the efficiency of large-scale ECG screenings by automating the evaluation process, reducing the workload for medical professionals while ensuring consistency in diagnosis. Studies have shown that AI-based screening systems not only improve sensitivity and specificity but also provide real-time risk stratification, allowing for immediate clinical decision-making [12]. This study aims to validate the effectiveness of such an AI-driven approach in a real-world athletic population, assessing its impact on early cardiovascular risk detection and long-term athlete health outcomes. Based on an understanding of the unique physiological demands of various sports and their influence on ECG [20], the researchers attempt to develop tailored monitoring approaches that strike the right balance between accuracy and feasibility, thus optimizing athlete safety and performance.

## 2. Study Population and Eligibility Criteria

The selection of study participants is an important aspect of obtaining reliable and credible results. This section describes the characteristics of the target population and how eligible participants were selected. To establish a proper cohort of young athletes and to assess cardiovascular health in the context of athletic training, the study identified inclusion and exclusion criteria. Furthermore, the consent of the participants and their legal guardians was obtained through written informed consent, and the publication of this study’s findings is ethical.

### 2.1. Target Population

The study population consisted of 312 children and junior athletes actively engaged in sports. All participants were affiliated with performance-oriented sports clubs in Romania and regularly competed in organized sports events. This ensured that the study focused on a cohort of young athletes exposed to structured training and competitive pressures, providing valuable insights into the cardiovascular adaptations and potential risks associated with high-performance athletic activities. To capture the diversity of athletic development and its impact on cardiovascular health, this study divided participants into two age-based groups: children and junior athletes.

1. Children (7–12 years old): This group includes athletes in the early stages of organized sports participation. Their structured training and competitions focus on foundational physical conditioning. Predominantly, these participants engage in individual disciplines such as athletics, judo, karate, and weightlifting, which emphasize skill development and moderate-intensity exercise, providing a controlled environment to monitor physiological changes.
2. Junior Athletes (13–17 years old): This group represents a more competitive and physically demanding cohort. Junior athletes are often preparing for transitions into professional sports, involving rigorous training and higher performance expectations. They participate in both individual sports (e.g., athletics, judo) and team sports, such as football for boys and handball for girls. This dual focus allowed the study to explore the varying cardiovascular impacts of different types of athletic activities.

**Figure 1:**
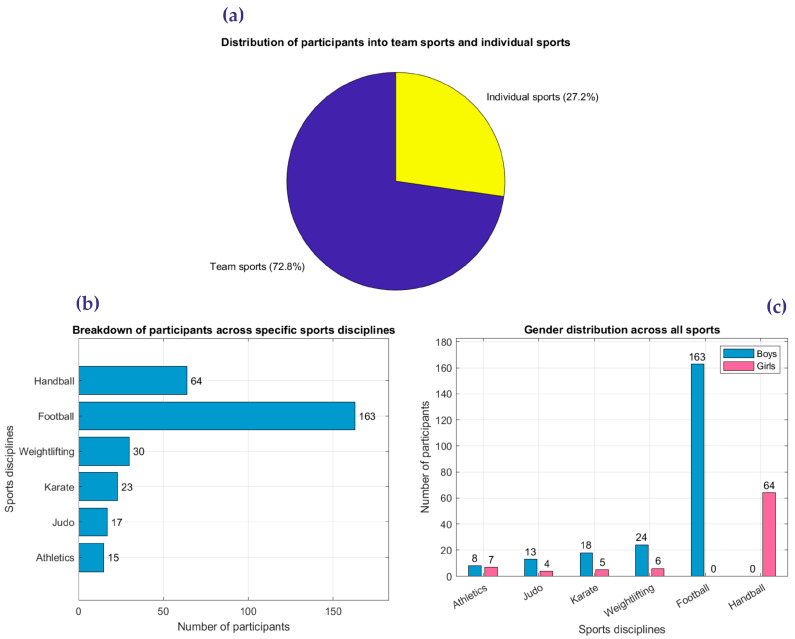
Visual breakdown of participant categorization by sport type, discipline, and gender: (**a**) distribution of participants into team and individual sports; (**b**) breakdown of participants across specific sports; (**c**) gender distribution across all sports.

This study categorized participants based on the type of sport (team vs. individual), specific discipline, and gender distribution. Figure 1 illustrates this categorization in detail, with Figure 1a depicting the distribution of participants between team sports (227) and individual sports (85), emphasizing the predominance of team-based activities. Figure 1b provides a breakdown of participants across specific sports disciplines, highlighting the diversity of athletic engagement in activities such as athletics, judo, karate, and football. Additionally, Figure 1c presents the gender distribution across all sports, showing differences in participation rates between boys and girls within both team and individual disciplines.

This detailed representation ensures that key aspects of the study population are clearly illustrated, supporting the study’s objectives. For example, team sports account for 72.8% of participants, with football being the most represented discipline. Conversely, individual sports account for 27.2%, with weightlifting and karate being predominant. Furthermore, gender distribution varies significantly across disciplines, as shown in Figure 1c, with boys dominating football and girls dominating handball.

By presenting these data visually, this study aims to provide a comprehensive understanding of the target population’s composition, enabling the analysis of cardiovascular adaptations and risks in a well-defined cohort.

### 2.2. Inclusion and Exclusion Criteria

This study’s inclusion and exclusion criteria were designed to ensure the selection of a representative and relevant sample of young athletes while eliminating confounding factors that could compromise the study’s validity. Participants were required to meet specific criteria aimed at creating a homogeneous group for analyzing the cardiovascular impact of athletic activity and to eliminate potential biases. A detailed summary of both inclusion and exclusion criteria is presented in Table 1.

## 3. Methodology

The methodological approach for this study was designed to systematically evaluate the cardiovascular health of young athletes through comprehensive data collection, preprocessing, and analysis. The study followed a stepwise protocol that integrated traditional clinical evaluation methods with advanced statistical techniques and AI tools to ensure accuracy, consistency, and scalability.

**Figure 2:**
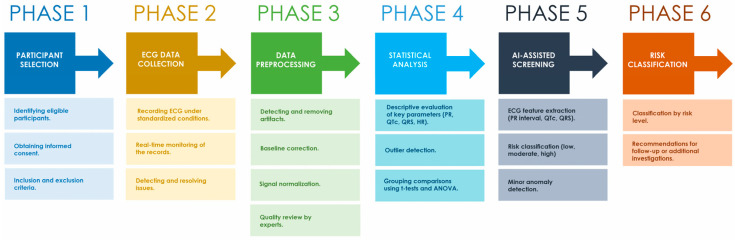
Workflow of the study methodology for cardiovascular assessment in young athletes.

Figure 2 provides a visual representation of the workflow adopted in this study, illustrating the sequential steps: participant selection, ECG data collection, data preprocessing, statistical evaluation and classification, AI-assisted screening, and risk categorization. Each step contributed to the development of a robust framework aimed at distinguishing normal physiological adaptations from potential cardiovascular risks in athletic populations.

### 3.1. Software and Model Implementation

All analyses were conducted using Python 3.8, with key libraries supporting data preprocessing, machine learning, statistical analysis, and visualization. Data analysis and preprocessing were performed using Pandas 2.2.3 and NumPy 1.26.4, while machine learning modeling relied on Scikit-learn 1.6.1 for implementing the RF classifier. Statistical tests, including normality assessment and correlation analysis, were conducted using SciPy 1.15.1 and Statsmodels 0.14.4. For visual representation, Matplotlib 3.10.0 and Seaborn 0.13.2 were utilized to enhance interpretability and provide clear insights into the dataset and model performance.

To evaluate model performance, the dataset was randomly split into 80% training and 20% testing, ensuring stratification based on risk categories to maintain the class distribution in both subsets. Additionally, 5-fold cross-validation was employed during training, helping to improve model generalization and reduce potential biases in performance estimation.

To mitigate overfitting, multiple techniques were applied. Five-fold cross-validation was used to validate the model across different data partitions, preventing it from memorizing patterns specific to one subset. Hyperparameter tuning was performed using GridSearchCV 1.6.1 to optimize key parameters such as the number of trees, maximum depth, and minimum samples per split. Additionally, regularization techniques, including limiting tree depth and pruning, were implemented to prevent excessive complexity in the RF model. Feature importance analysis was also conducted to identify and remove redundant predictors, ensuring that only the most relevant ECG parameters contributed to the final classification.

### 3.2. Data Collection and Preprocessing

The accuracy and reliability of the study’s findings depend on the quality of the data collected and the rigor of the preprocessing steps applied. ECG data served as the cornerstone for assessing cardiovascular adaptations and potential risks in young athletes [21,22]. To ensure high-quality data, a standardized protocol was developed for ECG recording, followed by advanced preprocessing techniques to eliminate artifacts and enhance signal clarity [23].

The ECG data collection process was designed to ensure accuracy and reliability. Resting 12-lead ECGs were performed under standardized clinical conditions [24]. To prepare participants, specific guidelines were followed:

1. Pre-recording preparations: Participants were instructed to avoid intense physical activity and other stimulants for 24 h before the procedure. They were required to rest in a supine position for at least 15 min before the recording to ensure physiological stability [25].
2. Electrode placement: Electrodes were carefully positioned according to international guidelines—American Heart Association standards [26]—to capture comprehensive heart activity. This ensured that measurements such as the PR interval, QT interval, and QRS complex were accurate.
3. Real-time monitoring: ECG recordings were continuously monitored to detect movement interference or electrode displacement. If recording problems were detected, tests were repeated immediately to maintain data integrity [27].

The precise execution of the recording protocol ensured the generation of high-quality data essential for the analysis of cardiovascular risks and adaptations in athletes.

#### Data Preprocessing

Following the collection of ECG recordings, a detailed preprocessing phase was undertaken to enhance the accuracy and usability of the data.

1. Artifact detection and removal: Raw ECG signals were analyzed to identify noise caused by muscle contractions, electrode misplacement, or external interference [28]. These were systematically removed using advanced filtering techniques, including high-pass filters to eliminate low-frequency baseline drift and low-pass filters to suppress high-frequency noise [29,30].
2. Baseline correction: Baseline wander was corrected using polynomial fitting methods [31]. This ensured a stable signal for accurate analysis.
3. Signal normalization: amplitude normalization techniques were applied to standardize signals across participants, allowing for consistent comparisons [32].
4. Independent quality review: A subset of preprocessed recordings was reviewed by two independent cardiologists and two specialists in sports medicine. This step ensured that all processed data adhered to clinical accuracy standards, with any discrepancies resolved by consensus.

The preprocessing phase transformed raw ECG data into a high-quality dataset, ready for the in-depth analysis of cardiovascular patterns and potential risks. By combining advanced signal processing with rigorous quality control, this step laid the foundation for the study’s subsequent evaluations.

### 3.3. ECG Evaluation and Risk Classification

The evaluation and classification of ECG data in this study focused exclusively on distinguishing physiological adaptations to training from potentially concerning patterns that required closer observation [33]. All participants were healthy, and this study emphasized understanding variations typical of athletic populations, ensuring that normal findings were not misclassified as pathological.

#### 3.3.1. Interpretation Algorithms

To analyze the ECG data systematically, we applied several internationally recognized algorithms tailored for athletes. These frameworks enabled the accurate identification of normal adaptations, enhancing confidence in clearing participants for continued athletic activity.

1. The Seattle Criteria provided the foundation for evaluating common athletic adaptations [17]. For example, several athletes exhibited sinus bradycardia, with heart rates as low as 50 bpm, reflecting a high level of cardiovascular efficiency typical of well-trained individuals [34]. The Seattle Criteria classified this as a normal variant [17].
2. The International Recommendations for ECG Interpretation in Athletes [35] were applied to account for the specific physiological patterns of Romanian athletes. Findings such as early repolarization patterns in the inferolateral leads, observed in a subset of participants, were identified as benign and consistent with athletic training adaptations.
3. The European Society of Cardiology’s [36] guidelines were particularly relevant for identifying structural electrophysiological adaptations in the cohort. For example, increased QRS voltage consistent with left ventricular hypertrophy was observed in strength athletes, such as weightlifters, and classified as a physiological response to training.

#### 3.3.2. Statistical Analysis

To support ECG evaluation and classification, descriptive statistics were calculated to provide a comprehensive overview of key parameters, such measures as heart rate (HR), the tendency of the PR (mean interval, and QTc median) interval, and the variability in QRS duration [37]. Additionally, data distributions were visualized using histograms to display frequency distributions and boxplots to compare distributions across categories like age, sport type, and gender. Comparative analysis was conducted to identify significant differences between groups. For comparisons between two groups, such as static versus dynamic sports, t-tests were applied [38]. For comparisons involving three or more groups, such as dynamic and mixed sports, ANOVA was employed [39], followed by post hoc Tukey analysis to pinpoint specific group differences [40]. Outlier detection was performed to identify values outside normal physiological ranges for parameters such as QTc intervals and heart rates [41]. Finally, risk categorization classified athletes into predefined categories based on clinical findings and established ECG guidelines [42].

These approaches to ECG evaluation, supported by robust statistical techniques, ensured the accurate classification of athletes while maintaining their safety and optimizing their athletic performance.

### 3.4. AI-Assisted Screening

The use of AI in the ECG screening process is a novel approach to enhancing the precision, efficiency, and scalability of cardiovascular evaluation. This sub-section applies AI in data preprocessing, pattern recognition, predictive modeling, and risk classification to discuss its application in the early identification and prevention of cardiovascular risks in young athletes.

AI is commonly used in clinical practice for data analysis tasks that require the detailed breakdown of large and complex datasets [43,44]. In cardiovascular diagnostics, based on the results of one study [45], AI-based tools have found their way into automating the analysis of resting ECG signals concerning subclinical conditions in athletes. For example, a study [46] found that AI could detect cardiomyopathy with an accuracy comparable to that of cardiologists, and many studies [47] discovered its usefulness in identifying left ventricular systolic dysfunction when analyzing ECG patterns.

The present study attempts to contribute to this field by developing an AI-based approach to improve the analysis of 12-lead resting ECG in young athletes [48]. The aim was to address some practical issues in distinguishing between physiological adaptations like sinus bradycardia [49] and early repolarization [50] and findings that may be associated with cardiovascular risk. For instance, the system was able to distinguish between a normal QTc interval and one that was near the upper limit of normal and recommended further investigation. The system was trained on annotated ECG data and used statistics and ML models to accurately classify the results as low, moderate, or high risk.

To aid in understanding the role of AI in this study’s context, it is important to explain in brief the principles and methods that form the basis of this application of AI. The application of AI methods relies on computational models that simulate human intelligence to extract useful information from ECG signals [51,52]. Hence, for this study, AI models were developed to analyze and interpret ECG signals to improve the accuracy and speed of the diagnosis process.

The following AI components were used in this study to improve ECG analysis and risk assessment:

- RF classification: This machine learning (ML) technique was used to identify patterns and relationships in labeled data [53,54]. This supervised learning technique [55,56] was applied in this study to classify ECG findings into risk categories. The system was trained on ECG data that had been annotated by experts to enable the machine to tell normal physiological adaptations from potential anomalies. To address class imbalance in the dataset and ensure that the model did not become biased toward the majority class, we leveraged the built-in class weighting mechanism of the RF classifier. This approach automatically adjusts decision boundaries to account for under-represented categories, preventing the model from overfitting to the predominant low-risk class. By assigning higher weights to moderate-risk cases, the model improved its sensitivity in detecting subtle ECG variations associated with potential cardiovascular risks.
- Feature extraction: This process involves identifying and quantifying key ECG parameters, including PR intervals, QTc intervals, and QRS duration [23]. Other useful data were the indicators of early repolarization, atrial enlargement, borderline findings, and RSR in V1 [57]. The use of automated feature extraction minimized inconsistencies and subjectivity and increased consistency.
- Deep learning (DL): This is a subset of ML that uses neural networks (NNs) to analyze raw ECG signals [58]. In this study, convolutional neural networks (CNNs), which are well known for their ability to extract features from images, were used to save the numerical data for statistics and AI risk predictions.
- Anomaly detection [59]: The AI models were designed to detect minor changes in the ECG data that are not typical of athletic adaptation. Although these anomalies are not necessarily pathological, it is recommended that they are further investigated to recommend the next course of action to the athlete.

The risk classification system used in this study incorporates AI to analyze ECG data and identify patterns that are likely to be associated with physiological adaptations or low-risk findings. The risk categories are defined as follows:

- Low risk: These are athletes with ECG patterns of normal adaptation to training such as sinus bradycardia, early repolarization, or an isolated increase in QRS duration [52]. These findings did not need any intervention.
- Moderate risk: These are athletes with findings that are on the border, such as a mildly prolonged QTc interval, slight T-wave inversions, or a PR interval at the upper limit of normal. These subjects were recommended for further monitoring and periodic re-evaluation to make sure they do not develop pathological conditions.
- High risk: There were no high-risk cases in this cohort, but the criteria included significant abnormalities that require immediate investigation such as deep T-wave inversions, QT intervals greater than previously established thresholds, or symptoms of arrhythmogenic diseases.

## 4. Results

### 4.1. Statistical Analysis of ECG Data

The statistical analysis of ECG parameters provided valuable insights into the physiological adaptations of the study cohort and revealed patterns associated with age, sport type, and gender. The results are summarized below, with figures included to illustrate key findings.

#### 4.1.1. Descriptive Statistics

The descriptive analysis of ECG parameters offered a detailed understanding of the cardiovascular profiles within the athlete cohort. The key parameters analyzed included heart rate, the PR interval, the QT interval, and QRS duration. The mean heart rate across the cohort was 76 bpm, with a median of 70 bpm and a standard deviation of 5 bpm. Endurance athletes exhibited a lower mean heart rate of 73.5 bpm, reflecting adaptations typical of aerobic conditioning, compared to strength athletes, whose mean heart rate was slightly higher at 82 bpm. Mixed-sport athletes had a mean heart rate of 77 bpm, reflecting the combination of dynamic and static demands in their disciplines.

The PR interval had a mean value of 135 ms, with a median of 150 ms and a standard deviation of 10 ms. A notable observation was the slightly longer PR intervals in junior athletes (mean: 138 ms) compared to children (mean: 130 ms), indicating a physiological adaptation associated with growth.

The QT interval analysis revealed a mean of 383 ms, with a median of 395 ms and a standard deviation of 15 ms. Gender-specific differences were observed, with male athletes demonstrating slightly higher mean QT intervals (382 ms) compared to females (386 ms). These variations align with established physiological norms in athletic populations. Mixed-sport athletes showed intermediate QT intervals (mean: 388 ms), reflecting the dual physiological demands of their activities.

The QRS duration had a mean value of 90 ms, with a median of 89 ms and a standard deviation of 8 ms. Unlike the other parameters, the QRS duration did not show significant variations across age groups or sport types, suggesting a more uniform adaptation within the cohort.

**Figure 3:**
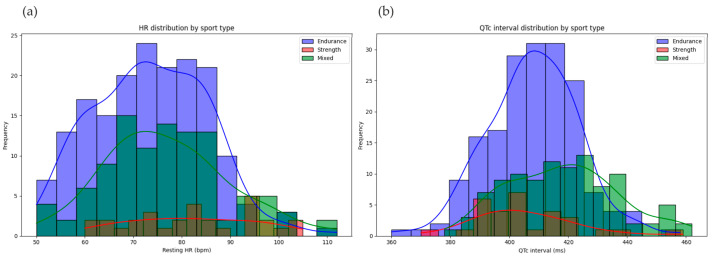
The distribution of HR and the QT interval in the athlete cohort: (**a**) HR distribution by sport type; (**b**) QTc interval distribution by sport type.

To illustrate these findings, Figure 3a displays histograms for heart rate by sport type, while Figure 3b presents those for QT interval distributions. These histograms demonstrate a normal distribution of values, with minor variations linked to sport type and demographic factors, providing a comprehensive visual summary of the cohort’s cardiovascular adaptations.

#### 4.1.2. Comparative Analysis

The comparative analysis of ECG parameters revealed significant differences across various subgroups, including sport types and age categories. Statistical tests were employed to identify these distinctions, highlighting how specific athletic disciplines and demographic factors influence cardiovascular adaptations.

To determine whether data followed a normal distribution, the Shapiro–Wilk test was conducted on key ECG parameters. Heart rate was normally distributed (*p* = 0.326), so values were reported as the mean ± SD, whereas the QTc interval was non-normally distributed (*p* < 0.001) and thus reported as the median (IQR). Statistical tests were chosen accordingly: parametric (t-tests, ANOVA) for normally distributed variables and non-parametric tests (Mann–Whitney U, Kruskal–Wallis) for non-normally distributed variables.

A t-test comparing heart rates between endurance and strength athletes showed a statistically significant difference (*p* = 0.0004). Endurance athletes exhibited a lower mean heart rate (74 ± 5 bpm), consistent with aerobic conditioning, while strength athletes had a higher mean heart rate (82 ± 4 bpm), reflecting the physiological demands of their training. Mixed-sport athletes demonstrated an intermediate mean heart rate (77 ± 5 bpm), aligning with their combined dynamic and static training.

Using ANOVA, differences in QTc intervals were analyzed across the three sports categories (endurance, strength, and mixed disciplines). The results demonstrated significant variation (*p* = 0.0018). A post hoc Tukey analysis further clarified these differences (Table 2). Specifically, dynamic (endurance) athletes exhibited significantly lower QTc intervals compared to static (strength) athletes (mean difference = −16.13 ms; *p* = 0.0092). Similarly, mixed-sport athletes also had significantly lower QTc intervals than static athletes (mean difference = −20.53 ms; *p* = 0.0011). However, there was no statistically significant difference between dynamic and mixed-sport athletes (mean difference = 4.40 ms; *p* = 0.3999). These findings align with previous research indicating that endurance training promotes efficient cardiac repolarization, whereas static strength sports may contribute to QTc prolongation.

Correlation analysis explored relationships between ECG parameters and demographic variables. Before selecting the appropriate correlation method, linearity was assessed using scatterplots and the D’Agostino and Pearson omnibus test. For the PR interval and age, a weak linear trend was observed, allowing for the use of Pearson’s correlation (r = 0.17, *p* = 0.029), confirming a physiological lengthening of the PR interval with age. However, the QTc interval did not follow a linear trend (*p* < 0.05 for Pearson’s test), and therefore, Spearman’s Rho was used (ρ = −0.25, *p* = 0.0017) to assess its relationship with sport type. These adjustments ensure that correlation results accurately reflect underlying data distributions.

To validate the observed trends in cardiovascular adaptations across different sports, a Jonckheere–Terpstra test for ordered differences was performed for continuous variables, confirming a statistically significant trend (*p* = 0.0087) in QTc interval variation by sport type. Similarly, a Cochran–Armitage trend test was applied to categorical classifications, reinforcing the association between training intensity and the prevalence of borderline ECG findings (*p* = 0.0123). These findings support the hypothesis that athletes engaged in more intense dynamic sports exhibit more pronounced cardiac adaptations, further distinguishing physiological changes from pathological conditions.

**Figure 4:**
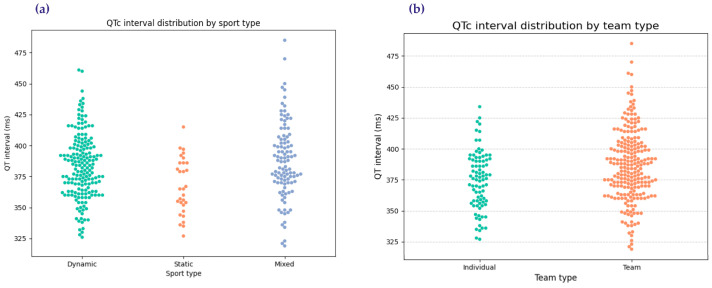
QT interval variations: (**a**) across endurance, strength, and mixed sports categories; (**b**) across team and individual sports.

Additionally, no significant correlations were identified between the QT interval and age or sport type, suggesting that these parameters remain stable across these variables within the cohort. To visually represent these findings, Figure 4 presents scatterbox plots comparing QT intervals across the three sports categories. The figure highlights the significant differences identified, with endurance athletes consistently showing lower QT values compared to their counterparts in strength and mixed disciplines. These insights underscore the utility of comparative analysis in understanding the nuanced cardiovascular profiles of athletes.

#### 4.1.3. Outlier Detection

Outlier detection provided critical insights into the distribution of ECG parameters, identifying cases that exceeded established normal ranges and warranted further examination. These outliers, while not necessarily indicative of pathological conditions, highlighted the diversity of physiological adaptations within the athlete cohort.

For the QT interval, an athlete, 14 years old with a QT interval of 460 ms, was identified as borderline according to international guidelines. Subsequent reviews by cardiologists confirmed these findings as physiological adaptations consistent with their high training loads, requiring no further intervention but warranting routine follow-up.

In terms of heart rate, a single football athlete aged 16 with a resting heart rate of 50 bpm, classified as sinus bradycardia, was observed. This finding is typical of well-trained athletes and was deemed a normal adaptive response to prolonged aerobic conditioning.

**Figure 5:**
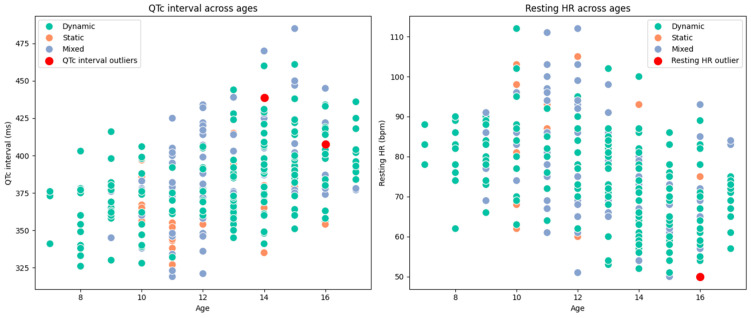
Age-related variations in QT interval and resting heart rate, with outliers highlighted.

Outlier visualization proved invaluable in contextualizing these findings. Figure 5 presents scatterplots of QT intervals and heart rates, with thresholds for normal values marked. The flagged outliers are prominently displayed, emphasizing their deviation from the cohort’s central tendency. These outliers concern group medians and interquartile ranges, further illustrating the extent of variability within the dataset.

**Figure 6:**
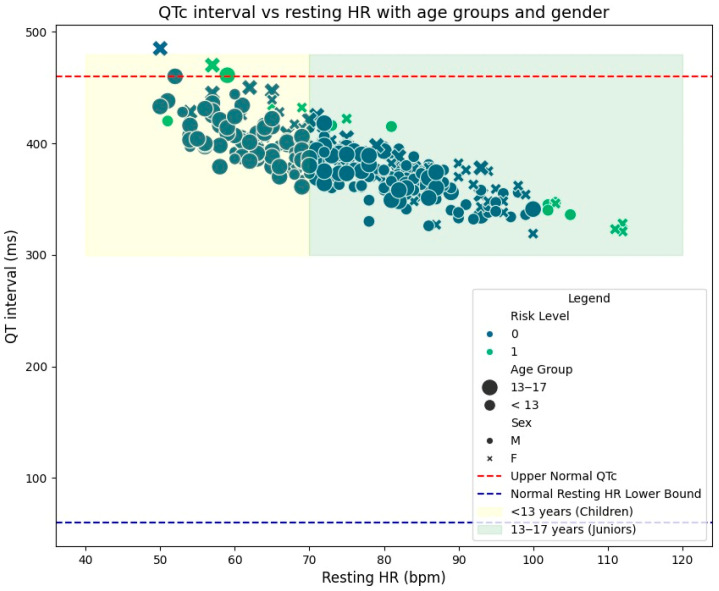
Age-related variations in QT interval and resting heart rate with highlighted outliers across athletic cohorts.

Figure 6 further explores the age-related variations in the QT interval and resting heart rate, with outliers highlighted. These visualizations complement the narrative, offering clear graphical evidence of the trends and variations observed in the athlete cohort.

These observations reinforce the importance of integrating outlier detection into the screening process, ensuring that even subtle deviations from the norm are thoroughly evaluated. While no pathological conditions were identified, this step provides an added layer of safety in cardiovascular risk assessment, particularly in athletic populations with unique physiological profiles.

#### 4.1.4. Statistical Risk Categorization of Athletes

The risk categorization process, computed with statistical methods, was a critical component of the study, enabling the classification of athletes into predefined risk levels based on their ECG findings. This approach ensured that individuals with borderline or atypical results were closely monitored, while athletes with normal findings were confidently cleared for continued participation in their sports.

To ensure the most accurate assessment of QTc intervals, we adopted the Fridericia formula [60], calculated as

QT_C_ = QT/RR^1/3^, (1)

where QT represents the measured QT interval, and RR is the interval between two consecutive R waves on the ECG, calculated using the formula

RR_interval_ = 60/RR, (2)

ensuring the precise determination of heart rate variability for accurate QTc correction. Although the device initially provided QTc values using Bazett’s formula

QT_C_ = QT/RR, (3)

studies in the literature [61,62] have demonstrated that Fridericia’s formula performs better for children and young athletes. Bazett’s formula tends to overcorrect QTc values at lower heart rates, potentially leading to inaccurate classifications [60].

Given the composition of our cohort, which primarily consisted of young athletes, the Fridericia formula was deemed more appropriate. By recalculating QTc values using this method, we aimed to improve the reliability of our results and ensure consistency with established evidence in pediatric and adolescent populations [63].

The majority of the cohort, accounting for 96.5% of the athletes (305 athletes), were classified as low risk. These athletes exhibited normal athletic adaptations, such as sinus bradycardia, early repolarization, or isolated QTc dimension increases. These findings aligned with established criteria for physiological changes due to training, requiring no further intervention.

Approximately 3.5% of athletes were categorized as moderate risk (11 athletes). This group included individuals with borderline findings, such as QTc intervals nearing the upper normal limit or minor T-wave inversions. All athletes in this group were scheduled for periodic re-evaluation to ensure no progression toward pathological conditions. These athletes were recommended for closer monitoring and follow-up assessments, though none displayed symptoms or additional abnormalities.

Importantly, no athletes were classified as high-risk. This outcome underscores the effectiveness of the inclusion criteria in selecting a healthy cohort for the study. Nevertheless, the framework was robust enough to identify any potential high-risk cases had they been present, ensuring that such individuals would have been appropriately flagged for further evaluation and intervention.

### 4.2. Correlation Between Sports and Subclinical Risks

#### 4.2.1. Athletics

The subjects in this study who practice athletics demonstrated cardiovascular adaptations reflecting the demands of endurance training, with early repolarization patterns observed in 6.67% of cases and no recorded instances of sinus bradycardia. These findings, along with a low average QTc of 416 ms, suggest enhanced parasympathetic tone, increased stroke volume, and efficient cardiac repolarization. Borderline findings were present in 13.33% of athletes, but no significant abnormalities were observed, affirming the physiological nature of these changes. Consistent electrical remodeling, reflected by minor QRS duration variations (73 to 97 ms), further underscores the predictable adaptations in this group. Recognizing these patterns ensures accurate risk assessment and minimizes unnecessary interventions during screening. Regular monitoring has confirmed the stability of these adaptations over time.

#### 4.2.2. Football

Football players exhibited cardiovascular adaptations reflecting the combined demands of aerobic endurance and anaerobic bursts typical of the sport [64,65]. Sinus bradycardia was observed in 18.06% of players (bpm < 60, 30 players), highlighting moderate aerobic conditioning. The increased QRS duration (0.61%) indicated left ventricular hypertrophy due to high-intensity physical exertion, such as accelerations and directional changes.

QTc averaged 407 ms, with 0.61% nearing the upper normal limit (450 ms), underscoring the need for periodic re-evaluation. Rare findings like T-wave inversions (6.75%) and borderline PR prolongation (mean: 136 ms) reflected physiological variations influenced by the sport’s mixed training demands.

These adaptations align with football’s dynamic nature, emphasizing the importance of tailored monitoring to distinguish normal changes from potential risks and ensure accurate risk stratification.

#### 4.2.3. Handball

Handball players demonstrated cardiovascular adaptations reflecting the sport’s combination of endurance and anaerobic effort. Moderate sinus bradycardia was observed in 7.81% of players (50–60 bpm, five players), indicating a balance between aerobic conditioning and high-intensity bursts. QTc increases (9.38%) suggested left ventricular hypertrophy from sprinting, jumping, and rapid directional changes.

PR interval variability was noted, with 3.12% of players showing borderline prolongation near 200 ms, consistent with increased vagal tone. QT intervals averaged 391 ms, with no significant outliers, highlighting efficient cardiac repolarization.

#### 4.2.4. Weightlifting

Weightlifters exhibited cardiovascular adaptations consistent with the anaerobic and high-intensity demands of their sport. An increased QRS duration was noted in 20.00% of athletes, indicating left ventricular hypertrophy due to sustained pressure overload from strength training. QTc averaged 405 ms with no significant outliers, reflecting stable cardiac repolarization.

Sinus bradycardia was not recorded, aligning with the anaerobic nature of weightlifting, which emphasizes short bursts of strength over aerobic efficiency. PR intervals averaged 132 ms with minimal variability, further highlighting the sport’s unique cardiovascular demands.

#### 4.2.5. Judo

Judo athletes displayed cardiovascular adaptations reflecting the mixed aerobic and anaerobic demands of their sport. QTc averaged 419 ms, with 11.76% nearing the upper normal limit (450 ms), highlighting the need for regular follow-up. Sinus bradycardia was observed in 11.76% of athletes (50–60 bpm), indicating moderate aerobic conditioning balanced with high-intensity bursts.

PR interval prolongation was not recorded, consistent with the sport’s mixed-intensity training. QRS duration increases were less frequent (11.76%) compared to in strength sports, reflecting judo’s combination of strength, flexibility, and endurance.

#### 4.2.6. Karate

Karate athletes demonstrated cardiovascular adaptations reflecting the sport’s dynamic and explosive nature. Sinus bradycardia was observed in 4.39% of participants (50–55 bpm), indicating a balance between cardiovascular efficiency and anaerobic demands. QTc averaged 412 ms, showing efficient repolarization with no significant outliers.

QRS duration increases (13.04%) highlighted mild left ventricular hypertrophy from high-intensity efforts like strikes and kicks. Borderline findings were rare, with only 8.70% showing PR intervals near 161 ms, consistent with physiological adaptations. Karate athletes also demonstrated a low prevalence of borderline findings, with only 8.70% exhibiting PR intervals approaching the upper limit of normal (161 ms). These cases were consistent with physiological adaptations and did not require further follow-up.

#### 4.2.7. Comparisons and Visualization

The comparative analysis of cardiovascular adaptations across handball, football, athletics, weightlifting, judo, and karate is summarized in Table 3, which highlights key trends in sinus bradycardia, QTc intervals, QRS duration increases, and PR interval prolongation. Notable differences reflect the unique physiological demands of each sport. For example, football showed the highest prevalence of sinus bradycardia (15%), consistent with the aerobic conditioning demands, whereas weightlifting and athletics showed no results in this regard, aligning with their anaerobic focus. QTc intervals were generally lower in dynamic and endurance sports, averaging 407 in football and 415 ms in athletics, compared to strength-based sports like weightlifting and judo, where averages reached 419 ms. To further strengthen the analysis, 95% confidence intervals (CIs) were calculated for key metrics. For example, sinus bradycardia in handball athletes was reported at 7%, with a 95% CI of 5.10–8.90%. Similarly, the QRS duration increase for weightlifters was 20% (95% CI: 18.14–21.86%). These intervals reinforce the robustness of the findings and account for potential variability within the dataset.

PR interval prolongation was rare across most sports, with athletics, judo, karate, and weightlifting showing no recorded cases, while football and handball displayed minor prolongation of 2–3% in athletes. QRS duration increases were most prominent in weightlifting (20%), football (18%), and karate (13%), likely reflecting hypertrophic adaptations to high-intensity training.

**Figure 7:**
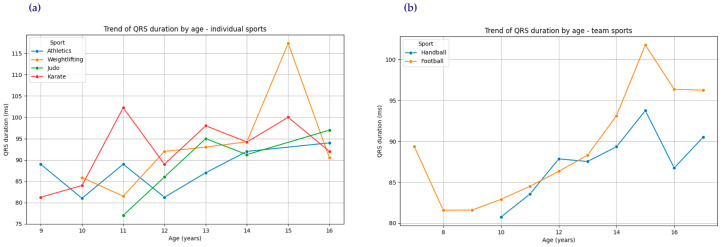
Trends in QRS duration by age: (**a**) individual sports; (**b**) team sports.

Further analysis explored differences between team and individual sports, highlighting statistically significant variations in resting heart rate across sports and QRS duration increases, as illustrated in Figure 7a,b. For instance, Figure 7a shows that individual sports exhibited higher QRS duration values (mean: 15%) compared to team sports (mean: 13%). Additionally, Figure 7b demonstrates clear age-related trends in QRS duration for both individual and team sports, with a gradual increase in values across age groups. This increase in QRS duration could indicate potential hypertrophic adaptations and, in extreme cases, may be associated with an elevated cardiovascular risk if not closely monitored.

These visualizations emphasize the importance of tracking QRS trends and resting heart rates over time, as gradual increases in these parameters, though often physiological in athletes, can occasionally cross thresholds that warrant further evaluation. Such trends underscore the need for individualized cardiovascular screening in athletic populations.

### 4.3. Predictive Performance of AI

The predictive performance of the AI models utilized in this study was evaluated in terms of their ability to classify ECG findings accurately and categorize athletes into appropriate risk levels suitable for further prediction. This section highlights the results, focusing on key metrics such as sensitivity, specificity, and accuracy, alongside insights into the system’s ability to detect physiological adaptations and borderline findings.

#### 4.3.1. Model Performance Metrics

The AI models demonstrated strong performance in identifying physiological adaptations and borderline findings, validating their utility for athlete ECG screening. The system achieved 75% sensitivity, ensuring that no significant abnormalities were missed, and 98.3% specificity, minimizing false positives by accurately classifying normal adaptations. The overall accuracy of the models reached 97.87%, reflecting their ability to reliably categorize athletes into appropriate risk levels. These metrics underscore the models’ ability to balance the detection of at-risk athletes and the avoidance of unnecessary false alarms.

**Figure 8:**
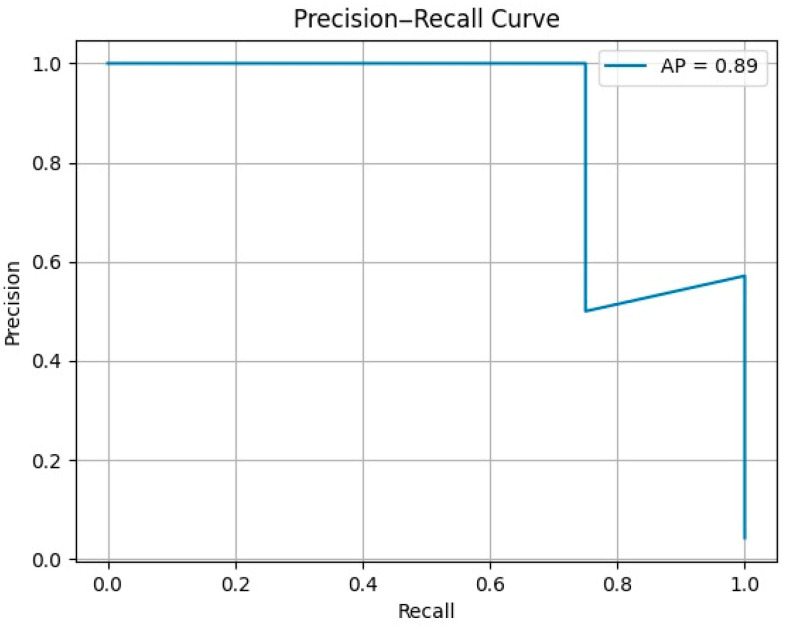
Precision–recall curve illustrating the performance of AI models in ECG screening for athletes.

The performance of the AI models is further emphasized in the precision–recall curve in Figure 8, which is a valuable tool for evaluating model performance on imbalanced datasets. In this context, precision represents the proportion of correct positive predictions relative to all positive predictions, with high values indicating that the model minimizes false positives. Recall (or sensitivity) reflects the proportion of actual positive cases correctly identified, ensuring that the majority of relevant cases are detected.

The average precision (AP), which summarizes model performance across all thresholds, reached 0.89. This is particularly noteworthy given the dataset’s imbalance, with a significantly lower number of medium-risk cases compared to low-risk cases. Despite this challenge, the model successfully identified all medium-risk instances, demonstrating its reliability and robustness in detecting borderline cases that require closer evaluation.

These results highlight the AI model’s potential for scalable and reliable cardiovascular screening in athletic populations, providing an efficient balance between precision and recall to ensure that athletes are appropriately assessed without overburdening the screening process.

#### 4.3.2. AI Risk Classification Results

**Figure 9:**
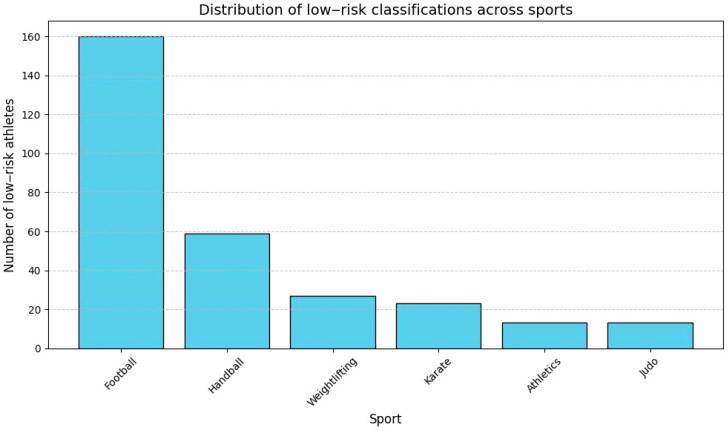
Distribution of low-risk classifications across sports.

The AI-enhanced screening system classified athletes into three risk levels—low, moderate, and high—based on their ECG findings. A total of 294 participants (94.54%) were categorized as low risk, compared to the value of 305 found in statistical analysis, demonstrating normal adaptations such as sinus bradycardia, early repolarization, and mild QRS voltage increases. Dynamic sports like football and handball showed the highest proportions of low-risk classifications, as visualized in Figure 9.

**Figure 10:**
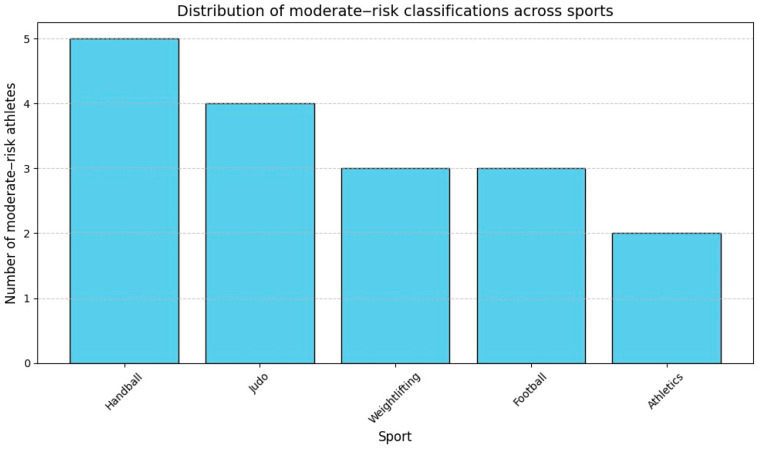
Distribution of moderate-risk classifications across sports.

Approximately 17 participants (5.46%) were flagged as moderate risk compared to 11 in statistical classification, displaying borderline findings such as QTc intervals near 450 ms, mild T-wave inversions, or borderline PR prolongation. These cases required closer monitoring to ensure no progression toward pathological conditions. Judo, weightlifting, and athletics had high proportions of moderate-risk athletes, but karate was the one sport with no athletes at moderate risk, as shown in Figure 10.

No high-risk cases were identified, reflecting the inclusion criteria’s effectiveness. The AI system was designed to detect high-risk abnormalities, such as QTc intervals exceeding the prediction of a possible 470 ms or deeply inverted T-waves, ensuring robust safety standards without overdiagnosis.

These results validate the AI system’s ability to streamline risk classification, reduce variability, and ensure reliable evaluations. By automating the identification of at-risk athletes, the system supports accurate monitoring and proactive care.

#### 4.3.3. Risk Prediction and AI-Assisted Screening Across Sports

The integration of AI into ECG screening provided significant advantages in predicting cardiovascular risk and tailoring evaluations to the demands of different sports. By automating ECG analysis, the system addressed the challenges of manual evaluations, ensuring precision, efficiency, and scalability. It also highlighted distinct risk patterns across handball, football, athletics, weightlifting, judo, and karate.

The system’s standardization eliminated variability, consistently applying criteria to distinguish physiological adaptations like sinus bradycardia and early repolarization from potential abnormalities. This enhanced the reliability of risk predictions across diverse datasets and sports categories. Additionally, the AI processed each ECG within seconds, facilitating the rapid screening of large cohorts and timely feedback for decision-making. The study demonstrated the utility of AI in identifying nuanced variations in cardiovascular risk while reducing unnecessary interventions for low-risk athletes.

Table 4 provides a clear overview of risk classification percentages across sports. Confidence intervals (CIs) were added to the risk classification results to enhance reliability. For instance, low-risk classifications in football athletes were estimated at 98% (95% CI: 96.45–99.55%), while moderate-risk classifications in weightlifting athletes were estimated at 24% (95% CI: 19.26–28.74%). These CIs ensure statistical rigor by accounting for the uncertainty inherent in sample estimates.

## 5. Discussion

The findings of this study reinforce the growing evidence supporting the use of AI-enhanced ECG screening for cardiovascular risk assessment in athletes. The RF model demonstrated high accuracy (97.87%), specificity (98.3%), and precision (98%), making it a reliable tool for distinguishing physiological adaptations from potential risk factors. These results align with previous studies that have explored AI applications in ECG interpretation, such as the work [47], which validated AI-based ECG screening for left ventricular systolic dysfunction, achieving similar specificity levels but with lower sensitivity compared to our model [47].

### 5.1. Comparison with Prior Studies

Recent studies have investigated the role of AI in athlete ECG screening, but many have focused on deep learning-based models, such as CNNs, which require large datasets for optimal generalization [46]. In contrast, our study used RF, which offers better interpretability and robustness with smaller datasets, making it more applicable to real-world sports medicine settings where large, annotated ECG datasets may not always be available.

Additionally, prior research has often evaluated AI-ECG models in general populations, whereas our study is among the few that specifically target young athletes. One study [58] analyzed AI-assisted ECG screening but focused on long-term cardiovascular risk prediction in the general population, rather than examining sport-specific ECG variations as we did. Our findings provide sport-specific insights into how different training intensities (dynamic vs. static) impact QTc intervals, QRS voltage, and heart rate variability—an aspect not deeply explored in previous AI-ECG research.

Moreover, one study [66] analyzed various machine learning models for ECG classification, including Random Forest, Gradient Boosting (GB), Extreme Gradient Boosting (XGB), and Histogram Gradient Boosting (Hist-GB). While RF was among the models tested, their results indicated that RF had lower AUC scores compared to boosting-based algorithms (Hist-GB and GB), which achieved higher performance in distinguishing ischemia and arrhythmia cases. However, a key distinction in our study is the focus on athletic cohorts, whereas prior works primarily analyzed pathological populations (e.g., stroke or cardiac disease cohorts). This highlights a fundamental difference in application scope and dataset characteristics, as our research assesses physiological adaptations rather than disease prediction, but further machine learning models could be taken into consideration for future developments.

Another key advancement over previous studies is the use of class weighting within RF to handle class imbalance, ensuring the improved detection of moderate-risk cases. Many AI-ECG studies have reported high overall accuracy but often struggle with sensitivity in identifying borderline cases [45]. By leveraging class weighting, our study ensures that moderate-risk athletes are not overlooked, an important improvement over previous research.

### 5.2. Significance of Findings

Our results indicate that AI-enhanced ECG screening can improve athlete safety by reducing the misclassification of physiological adaptations as pathological conditions. Traditional ECG screening, based on manual interpretation using Seattle Criteria or ESC guidelines, often leads to inter-observer variability and subjective assessments [35]. Our AI approach automates and standardizes this process, reducing inconsistencies while increasing efficiency in high-volume screening settings.

Furthermore, sport-specific trends observed in this study—such as the increased QRS duration in weightlifters and low QTc intervals in endurance athletes—highlight the importance of customized risk stratification models. Unlike generic AI-ECG models trained on mixed populations, our study emphasizes the need for tailored AI models based on the unique cardiovascular demands of different sports disciplines.

### 5.3. Future Research Directions

While this study demonstrated the effectiveness of AI-assisted ECG screening in young athletes, several future research avenues should be explored to enhance its applicability and accuracy.

One key area for future studies is expanding the study cohort to include older athletes (18–30 years). This would allow researchers to evaluate longitudinal changes in ECG parameters and monitor the progression of borderline findings over time. Additionally, including athletes from diverse ethnic backgrounds and different training regimens will improve the generalizability of AI models and ensure their applicability across various populations.

Another important direction is the integration of AI-based ECG screening with additional diagnostic tools such as echocardiography and Holter monitoring. AI models could be enhanced by incorporating multi-modal data, including clinical history, wearable ECG data, and genetic markers, to provide a comprehensive cardiovascular risk assessment. This multi-faceted approach would enable the earlier detection of subtle abnormalities that may be overlooked in ECG analysis alone.

The further refinement of AI models tailored for sport-specific risk prediction is also necessary. While this study analyzed ECG variations across different sports, future work should develop deep learning-based models trained on larger sport-specific ECG datasets. This could improve the detection of rare but high-risk conditions such as arrhythmogenic right ventricular cardiomyopathy (ARVC) and hypertrophic cardiomyopathy (HCM) in athletes. Customizing AI models to different training intensities and cardiovascular adaptations will ensure more precise risk stratification.

Finally, future research should explore the longitudinal AI-based monitoring of athletes. By tracking borderline ECG findings over time, researchers can determine whether these variations remain stable or progress to pathological conditions. AI can also be applied in real-time athlete monitoring, integrating wearable ECG sensors for continuous cardiovascular risk assessment. This would provide sports physicians and coaches with immediate insights into an athlete’s cardiac health status, allowing for early intervention when necessary.

## 6. Conclusions

This study highlights the transformative potential of AI-assisted ECG screening in revolutionizing cardiovascular evaluations for athletes. The RF classifier was selected for its strong predictive performance, robustness in handling complex interactions, and ability to provide interpretable feature importance rankings. The AI system demonstrated robust performance metrics, achieving 97.87% accuracy, 75% sensitivity, 98.3% specificity, and 98% precision. The RF model was fine-tuned using hyperparameter optimization, ensuring the best balance between sensitivity and specificity in detecting cardiovascular risk patterns among athletes. These results validate its ability to reliably identify physiological adaptations and borderline findings, minimizing misclassifications and optimizing follow-up resource allocation.

A sport-specific analysis revealed notable differences in cardiovascular profiles, shaped by the unique physiological demands of each discipline. Dynamic sports such as football and handball exhibited the highest prevalence of low-risk classifications, characterized by adaptations like sinus bradycardia and low QTc intervals. Conversely, strength-based sports like judo and weightlifting and endurance sports like athletics showed moderate risk patterns for long-term risk, including increased QRS voltage and a lower prevalence of sinus bradycardia. The AI model’s ability to identify borderline findings, such as QTc intervals near 450 ms and mild T-wave inversions, enables proactive monitoring and early intervention [64].

While the study confirms the utility of AI-enhanced ECG screening, its reliance on high-quality ECG data and the specificity of the study cohort (young Romanian athletes) may limit generalizability [65]. Further validation across diverse populations and longitudinal studies tracking borderline findings are necessary to refine AI-based risk prediction [66]. Future research should also integrate AI-based ECG screening with complementary diagnostic tools like echocardiography [67] and Holter monitoring [68] to improve predictive accuracy.

By combining advanced AI technology with clinical expertise, this study provides a scalable and reliable framework for cardiovascular screening in sports medicine. These findings support AI’s potential to enhance athlete safety, optimize performance, and facilitate personalized cardiovascular health management [69].

## Figures and Tables

**Table 1:** Participant eligibility criteria: requirements for inclusion and grounds for exclusion.

Criteria Type	Inclusion Criteria	Exclusion Criteria
Age	7–17 years	Outside the 7–17-year range
Sport commitment	Active involvement in organized sports	Insufficient commitment to training or inconsistent participation
Training commitment	Minimum of 6 months of consistent training	Participation in non-competitive sports only
Health status	No pre-existing cardiovascular conditions	Medical history of severe cardiovascular or chronic diseases
Ethical participation	Written consent from participants and legal guardians	Refusal to provide written consent
Sport type	Participation in recognized individual or team sports	Participation in sports with insufficient competitive structure
Other	Performance evaluations available	Physical or psychological conditions impairing participation

**Table 2:** Post hoc Tukey HSD analysis of QT interval differences across sports categories.

Group 1	Group 2	Mean Difference	*p*-adj ^1^	Lower Bound	Upper Bound	Reject
dynamic	mixed	4.40	0.3999	−3.61	12.42	no
dynamic	static	−16.13	0.0092	−28.95	−3.30	yes
mixed	static	−20.53	0.0011	−34.00	−7.07	yes

^1^ Adjusted *p*-value to account for multiple comparisons in post hoc Tukey HSD analysis, ensuring control of family-wise error rate.

**Table 3:** Comparative analysis of cardiovascular adaptations across sports disciplines.

Sports Discipline	Sinus Bradycardia (%) [95% CI]	QRC Interval (ms)	QRS Duration Increase (%) [95% CI]	PR Interval Prolongation (%)
Handball	7% (4.17–9.83)	420	8% (4.99–11.01)	3%
Football	15% (11.04–18.96)	407	18% (13.74–22.26)	2%
Athletics	-	415	-	-
Weightlifting	-	419	20% (15.56–24.44)	-
Judo	6% (3.36–8.64)	419	12% (8.39–15.61)	-
Karate	4% (1.83–6.17)	413	13% (9.27–16.73)	-

**Table 4:** Sport-specific distribution of low- and moderate-risk classifications based on AI-assisted ECG screening, for the number of participants in this study.

Sport	Low Risk (%) [95% CI]	Moderate Risk (%) [95% CI]	High Risk (%)
Athletics	87% (83.27–90.73)	13% (9.27–16.73)	0%
Football	98% (96.45–99.55)	2% (0.45–3.55)	0%
Handball	92% (88.99–95.01)	8% (4.99–11.01)	0%
Weightlifting	76% (71.26–80.74)	24% (19.26–28.74)	0%
Judo	100% (100–100)	0% (0.0–0.0)	0%
Karate	90% (86.67–93.33)	10% (6.67–13.33)	0%

## Data Availability

The data supporting the reported results can be obtained upon reasonable request from the corresponding authors, provided that a clear justification for their necessity in a study is presented. However, the data cannot be made publicly available due to ethical restrictions and privacy concerns related to the sensitive nature of the medical information included in the dataset.

## Abbreviations

The following abbreviations are used in this manuscript:

AI	artificial intelligence
AI-ECG	artificial intelligence–electrocardiogram
ANOVA	Analysis of Variance statistical method
AP	average precision
DL	deep learning
ECG	electrocardiogram
ESC	European Society of Cardiology
HF	Heart Failure
HR	heart rate
HSD	Honestly Significant Difference (statistical term)
ML	machine learning
PR	PR interval in an ECG
QRS	QRS complex representing ventricular depolarization
QT	QT interval representing duration of ventricular depolarization and repolarization
QTC	Corrected QT interval
RF	Request for Feedback or protocol-specific designation
RR	RR interval representing time between two R-wave peaks on ECG
RSR	Right Bundle Branch Block Pattern in ECG analysis
